# Evaluation of physicochemical and mechanical properties of two experimental premixed calcium silicate sealers

**DOI:** 10.1590/0103-644020255937

**Published:** 2025-07-11

**Authors:** Maria Carolina Guiotti de Oliveira, Stefani Jovedi Rosa, Bruno Carvalho de Vasconcelos, Rodrigo Ricci Vivan, Murilo Priori Alcalde, Marco Antonio Hungaro Duarte

**Affiliations:** 1 Department of Dentistry, Endodontics and Dental Materials, Bauru School of Dentistry, USP, Bauru, São Paulo, Brazil; 2 Department of Dentistry, School of Dentistry of Sobral, UFC, Sobral, Ceará, Brazil

**Keywords:** physicochemical properties, tricalcium silicate, premixed sealer, bioceramic, root canal sealer

## Abstract

The study aimed to evaluate the physicochemical properties of two experimental root canal sealers (CEO 1 and CEO 2) and compare them with Bio-C Sealer (BC) and AH Plus Bioceramic (AHPB). Setting time was evaluated following ISO 6876 Standard, radiopacity was assessed by radiographic analysis in millimeters of aluminum, and flow was also evaluated following ISO 6876. Solubility was assessed through mass loss (%) after 7 days of immersion in distilled water, hydroxyl and calcium ions release was measured by pH-meter and atomic absorption spectrophotometer, respectively. Push-out was tested at the universal test machine. The data were statistically compared using a 5% significance level. CEO 1 and CEO 2 showed higher setting times (p<0.05) and all sealers demonstrated radiopacity higher than 3mm/Al; however, lower radiopacity and flow values were detected in the presence of both experimental sealers when compared with BC and AHPB (p<0.05). No difference was found among all sealers for solubility (p>0.05). BC and CEO 2 had higher pH values at the initial time (p<0.05) while AHPB at the final time (p<0.05). In general, no significant difference between all sealers at all times was observed, except at 168h for CEO 1, which released more calcium ions than CEO 2 (p<0.05). BC and AHPB provided a superior push-out (p<0.05), and cohesive failures were predominant for all sealers. Both experimental sealers exhibited physicochemical properties similar to commercial endodontics sealers.

## Resumo

O estudo teve como objetivo avaliar as propriedades físico-químicas de dois cimentos endodônticos experimentais (CEO 1 e CEO 2) e compará-las com Bio-C Sealer (BC) e AH Plus Bioceramic (AHPB). O tempo de presa foi avaliado seguindo o padrão ISO 6876, a radiopacidade foi avaliada através de análise radiográfica em milímetros de alumínio e o escoamento também foi avaliado seguindo a ISO 6876. A solubilidade foi avaliada através da perda de massa (%) após 7 dias de imersão em água destilada, a liberação de íons hidroxila e cálcio foi medida por phmetro e espectrofotômetro de absorção atômica, respectivamente. O push-out foi testado na máquina de testes universal. Os dados foram comparados estatisticamente utilizando nível de significância de 5%. CEO 1 e CEO 2 apresentaram tempos de presa maiores (p<0,05) e todos os cimentos demonstraram radiopacidade superior a 3mm/Al; entretanto, foram detectados menores valores de radiopacidade e escoamento na presença de ambos os cimentos experimentais em comparação com BC e AHPB (p<0,05). Não foi encontrada diferença entre todos os cimentos quanto à solubilidade (p>0,05). BC e CEO 2 apresentaram maiores valores de pH no momento inicial (p<0,05) enquanto AHPB no momento final (p<0,05). De modo geral, não foi observada diferença significativa entre todos os cimentos em todos os tempos, exceto às 168h para o CEO 1 que liberou mais íons cálcio que o CEO 2 (p<0,05). BC e AHPB proporcionaram push-out superior (p<0,05) e falhas coesivas foram predominantes para todos os cimentos. Ambos os cimentos experimentais exibiram propriedades físico-químicas semelhantes aos cimentos endodônticos comerciais.

**Figure:**
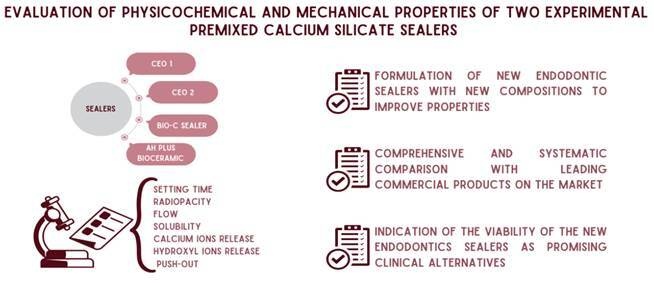

## Introduction

Calcium silicate-based sealers have gained attention because of their biocompatibility and capacity to set in humidity presence, forming hydroxyapatite crystals ^1^. These sealers are available in various formulations and configurations, including powder/liquid, two-paste resin systems, or single-paste incorporating organic liquids like Bio-C Sealer (BC) (Angelus Indústria de Produtos Odotontológicos S/A, Londrina, PR, BR) and AH Plus Bioceramic (AHPB) (Dentsply Sirona, Charlotte, NC, USA) ^2^.

BC is a tricalcium silicate sealer composed of tricalcium silicate, dicalcium silicate, tricalcium aluminate, calcium oxide, zirconia oxide, silicon oxide, polyethylene glycol, and iron oxide that exhibits low volumetric alteration, good alkalization, and flow capacity; however, it demonstrates high solubility ^3^. AHPB is a new tricalcium silicate sealer composed of zirconium dioxide, tricalcium silicate, dimethyl sulfoxide, lithium carbonate, and thickening agents ^4^. This sealer shows a faster setting time, lower film thickness, and higher radiopacity, although its solubility is a significant limitation, potentially adversely affecting obturation quality ^5^.

Despite these endodontic sealers demonstrating satisfactory biological and physicochemical properties ^6^, a lack of these properties still needs improvement since those are responsible for one of the principal roles in endodontic therapy success. Thus, developing new sealers that fill all these gaps is pertinent. In the composition of experimental sealers, the proportion of hydrated calcium silicate was reduced, monobasic calcium phosphate was added and zirconium oxide was combined with calcium tungstate. Additionally, new components, such as polyvinyl acetate, were incorporated into the composition of the liquid to improve plasticity and reduce solubility. So, this study aimed to evaluate the physicochemical properties of two experimental tricalcium silicate-based sealers (CEO 1 e CEO 2) and propose a new commercial biomaterial. The null hypothesis tested is that both experimental sealers present similar properties to endodontic sealers available BC and AHPB.

## Materials and methods

All materials tested in this study are described in Table 1. The sample calculation used G * Power v. 31 for Mac by selecting ANOVA fixing effects. The data from a previous study (Cavenago et al. ^7^ for the setting time, Duarte et al. ^8^ for the radiopacity, flow, solubility, pH, and calcium release, Silva et al. ^11^ for the push-out) were used. The effect size utilized in the present study was established (=0.80). The alpha-type error is 0.05, and the beta power is 0.90. A total of 03 specimens were necessary for each group for setting time, radiopacity, and flow, 05 specimens per group for the solubility, pH, and calcium release, and 10 specimens per group for the push-out.

**Table 1:** Composition of sealers tested and their manufacturers.

Sealer	Composition	Manufacturer
Bio-C Sealer	Tricalcium silicate, dicalcium silicate, tricalcium aluminate, calcium oxide, zirconia oxide, silicon oxide, polyethylene glycol, and iron oxide	Angelus, Londrina, PR, BR
AH Plus Bioceramic	Zirconium dioxide, tricalcium silicate, dimethyl sulfoxide, lithium carbonate and thickening agents	Dentsply Sirona, Charlotte, NC, USA
CEO 1	Tricalcium silicate, calcium oxide, calcium phosphate monobasic monohydrate, zirconium oxide, calcium tungstate and polyvinyl acetate	University of São Paulo, Bauru, SP, BR
CEO 2	Tricalcium silicate, calcium oxide, calcium phosphate monobasic monohydrate, zirconium oxide, calcium tungstate, calcium chloride and polyvinyl acetate.	University of São Paulo, Bauru, SP, BR

### Setting Time

The test was conducted under controlled temperature and humidity (37 °C ± 1 °C and 95% ± 5%). Type IV plaster rings (10 mm diameter and 2 mm thickness) were performed and immersed in deionized water for 24 hours. Then, the sealers were placed in rings and kept in an oven at 37^o^C. After 180 ± 5 seconds, the specimens were marked using a Gilmore needle with a weight of 113.5g to determine the initial setting time, and subsequently, a Gilmore needle weighing 456.3g was employed to determine the final setting time according to Cavenago et al. ^7^. 

### Radiopacity

For the radiopacity test, we used 3 specimens of each tested sealer. The sealers were placed in PLA (polylactic acid biopolymer) rings (10mm diameter and 1mm height) fabricated by a 3D printer (GTMAX 3D Core H4, GTMax3D, Americana, SP, BR) and stored in an oven at 37^o^C for setting. Then, the specimens were positioned on Kodak occlusal radiographic films (Kodak Comp, Rochester, NY, USA), and radiographed. An x-ray unit (Dabi Atlante, Ribeirão Preto, SP, BR) operating at 60 kV, 10 mA, and 0.3-second exposure time and focus-film distance of 30 cm was used. The images were processed, and digitalized and radiographic density values were evaluated following Duarte et al. ^8^. 

### Flow

For the flow test, 0.05 mL of each sealer was placed on the center of a glass plate, weighing 20 g and dimensions 40 mm and 5mm thickness, using a graduated syringe. After 180 seconds, a second glass plate of the same dimension was placed centrally on the sealer top, followed by a weight giving 120 g of total mass. Ten minutes after initiating mixing, the weight was removed, and the maximum and minimum diameters of compressed sealer discs were measured. The test was performed in triplicate ^4^.

### Solubility

This test was performed according to Tanomaru-Filho ^9^ with some modifications. We used 3 specimens of each tested sealer. Type IV plaster rings (20 mm diameter and 1.5 mm thickness) were confectioned and immersed in deionized water for 24 hours. Then, the sealers were placed in these rings with an impermeable nylon thread inserted inside the sealer mass and positioned between two glass plates with cellophane paper between them. Then, the set was taken to the oven (37 °C ± 1 °C and 95% ± 5%) for 3 times the duration of their setting time. Then, samples were removed from the rings and weighed on a precision scale. After, they were suspended by nylon thread and placed in sealed containers with 50 mL of deionized water. After seven days, samples were removed, dried with absorbent paper, dehumidified, and weighed again to determine the mass loss ^9^.

### pH levels and calcium ions release

Five polyethylene tubes (10mm length and 1mm diameter) were filled with the sealers. Specimens were placed in test tubes containing 10 mL of deionized water, and pH measurements were taken at 3, 24, 72, and 168h using a previously calibrated digital meter. After each period, the tubes were placed in a new flask with deionized water. The pH value of the water measured after each period was subtracted from the water pH before immersion, and the value of hydroxyl ions released was determined. After these measurements, the calcium released amount into deionized water was determined at 3, 24, 72, and 168h using an atomic absorption spectrophotometer (AA6800; Shimadzu, Tokyo, Japan) ^10^.

### Push-out

The local ethics committee approved this research (009/2023). Ten dentin discs (1.0 ± 0.1 mm thick) were prepared from bovine teeth using a low-speed saw (ISOMET, Buhler Ltd., Lake Buff, NY, USA) with a diamond disc of Ø 125 mm × 0.35 mm × 12.7 mm (Buhler Ltd.), under continuous irrigation. Then, four holes were drilled in each slice surface using a cylindrical carbide bur with 0.8 mm diameter and constant irrigation ^11^. Discs were immersed in a 2.5% sodium hypochlorite (NaOCl) solution for 15 min followed by immersion in bi-distilled water. The smear layer was eliminated using 17% EDTA applied for 3 minutes. Following this, the dentine discs were affixed to a glass plate and each cavity was filled randomly with the tested sealers. Subsequently, the filled discs were maintained in contact with sterile gauze moistened with PBS solution (pH 7.2) at 37 °C for 7 days before the push-out assessment ^11^. The push-out test was performed in a universal testing machine (Instron, Canton, MA, USA) with a plunger tip of 0.6 mm diameter that was positioned over only one of the tested materials for each analysis. The load was applied at 0.5 mm/min speed until failure. During load application, loading time (N) × displacement (mm) was recorded by a real-time software program, and bond strength was calculated in mega Pascal (MPa). Finally, the discs were examined under an INALH stereomicroscope (MSZ-300) at ×3 magnification to determine the bond failure mode, which was classified into three categories: (i) adhesive, (ii) cohesive, or (iii) mixed ^12^.

### Statistical analysis

Before the statistical analysis, all data obtained employing different evaluations were submitted to the Kolmogorov-Smirnov test to verify the standard distributions. Flow and setting times were analyzed by ANOVA, followed by Tukey’s tests. Radiopacity, solubility, pH level, calcium ion release, and push-out data were evaluated using the Kruskal-Wallis test and Dunn multiple comparison tests. Graph Pad Prism (version 9.0) software program was used for statistical analysis. The p-value was considered significant at 5%.

## Results

CEO 1 presented the most significant initial setting time (p<0.05). The final setting time of CEO 1 and CEO 2 was more significant than BC and AHPB (p<0.05), although CEO 1 demonstrated superior time than CEO 2 (p<0.05) (Table 2). 

**Table 2:** Mean and standard deviation of the radiopacity (mm Al), flow (mm), solubility (%), push-out (mPa) and setting time (min) of all sealers.

Sealers	Radiopacity (mm Al)	Flow (mm)	Solubility (%)
Bio-C Sealer	5.41 (1.66)^a^	28.25 (1.83)^ab^	19.02 (2.77)^a^
AH Plus Bioceramic	5.80 (1.22)^a^	31.62 (1.08)^a^	20.08 (2.3)^a^
CEO 1	3.18 (0.91)^b^	23.25 (3.98)^b^	49.20 (41.14)^a^
CEO 2	3.09 (0.27)^b^	23.22 (2.48)^b^	11.70 (12.91)^a^
Sealer	Push-out	Setting time (min)	
		Initial	Final
Bio-C Sealer	3.45 (1.9)^a^	107.6 (9.86)^a^	248.7 (30.07)^a^
AH Plus Bioceramic	3.3 (2.48)^a^	117 (27.52)^a^	275.5 (13.26)^a^
CEO 1	0.61 (0.8)^b^	201.7 (14.9)^b^	572.3 (43.25)^b^
CEO 2	0.63 (0.32)^b^	140.2 (6.49)^a^	477.8 (12.98)^c^

Different letters in each column indicate statistically significant differences (p<.05).

Concerning radiopacity, flow, and push-out tests, no difference was observed between experimental sealers (p>0.05); moreover, both experimental sealers exhibited values significantly lower than BC and AHPB (p<0.05). In addition, cohesive failures were predominant for all sealers evaluated. On the other hand, no difference was found among all sealers in terms of solubility (p>0.05) (Table 2).

BC had more hydroxyl ion release than AHPB at 3h and 72h (p<0.05) and CEO 1 at 3h, 72h and 168h (p<0.05). Besides, a lower hydroxyl ion release value was detected in the AHPB about CEO 2 at 24h (p<0.05). So, a comparison among the same sealer in time showed that BC and CEO 2 had higher hydroxyl ion release values at the initial time (p<0.05) while AHPB at 168h (p<0.05). CEO 1 had significantly more hydroxyl ion release at 24h than at 3h and 168h (p<0.05) (Table 3).

**Table 3:** Mean and standard deviation of hydroxyl released variation values and calcium ions released found for each sealer, in the different time intervals analyzed.

Sealers	pH values			
	3h	24h	72h	168h
Bio-C Sealer	2.93±1.02^cb^	1.75±0.94	1.62±0.05^b^	1.08±0.08^AB^
AH Plus Bioceramic	0.38±1.33	0.04±0.05	0.55±0.16	0.82±0.07^B^
CEO 1	0.83±0.42	1.95±0.09^A^	1.58±0.11^b^	0.63±0.1^aB^
CEO 2	1.36±0.35	2.29±0.2^b^	1.33±0.13	0.9 ± 0.12^B^
Sealers	Calcium íons			
	3h	24h	72h	168h
Bio-C Sealer	4.97±0.54	1.15±0.3^A^	4.34±0.42^B^	2.23±1.08^A^
AH Plus Bioceramic	2.85±1.57	2.5±1.51	3.54±1.27	3.29±1.92
CEO 1	4.23±0.77	1.9± 0.38	1.61±0.19^A^	5.20±1.63^C^
CEO 2	3.75±2.3	2.0±1.75	3.96±2.4	1.78±1.39^c^

Lower letters indicate differences intergroup observed in the comparison of different sealers at the same time: a: versus Bio-C Sealer; b: versus AH Plus Bioceramic; c: versus CEO 1. Capital letters indicate differences intragroup observed in the comparison of the same sealer at different times: A: versus 3h; B: versus 24h; C: versus 72h.

In general, no significant difference was detected for all sealers at times, except at 168h for CEO 1, which released more calcium ions than CEO 2 (p<0.05). Comparison between sealers at the same time showed that, at 3h, BC exhibited the highest calcium ion release when compared with 24h and 168h (p<0.05). In addition, a decrease at 24h and a raise at 72h was also observed in the presence of BC (p<0.05). Besides, the lowest CEO 1 calcium values were found at 72h when compared with 3h and 168h (p<0.05) (Table 3).

## Discussion

The physicochemical properties of endodontic sealers have a major impact on the obturation quality, independent of the technique used ^13^. Therefore, this study evaluated the physicochemical properties of two ready-to-use experimental sealers, and the null hypothesis was rejected since there were differences in any physicochemical properties. 

It has already been described that a prolonged setting time can raise material solubility and create gaps that may be critical for treatment success ^3^. In this investigation, both experimental sealers increased the final setting time compared to BC and AHPB. These results can be supported by the dispersing agent, which behaves just like propylene glycol, that can delay the setting time by reducing the amount for this reaction ^14^. By the way, CEO 1 demonstrated superior time over CEO 2, which can be explained by the calcium chloride present in CEO 2’s composition since it penetrates sealer pores, accelerating silicate crystallization and reducing setting time ^15^. The shorter setting times exhibited by BC and AHPB can also be explained by the fact that silicates and calcium aluminate generate hydrated by-products, resulting in a faster setting ^16^.

The radiopacity of an endodontic sealer must make it possible to differentiate it from the dentin or bone to verify the obturation quality ^17^ ^,^ ^8^. ISO standards ^17^ determine that obturation materials should exhibit a minimum radiopacity of 3 mm/Al, and all sealers demonstrated radiopacity above the recommended. However, both experimental sealers displayed significantly lower radiopacity values than BC and AHPB. Since the sealer radiopacity depends on the quantity and proportion of each radiopacifying agent, this difference may be attributed to incorporating zirconium oxide and calcium tungstate additives into the experimental sealer’s compositions ^8^. This finding corroborates Duarte et al. ^8^, who related the addition of calcium tungstate and zirconium oxide to Portland cement produced lower radiopacity values than those of bismuth oxide, lead oxide, iodoform, and bismuth. 

Flow is an essential property that allows sealers to fill areas of anatomical complexity; moreover, it is reported that excessive flow increases apical extrusion risk ^6^. All sealers evaluated exceeded the minimum value of 17 mm required by ISO ^17^, and the lowest values of AHPB were found in both experimental sealers. The experimental sealers also have polyvinyl acetate in their composition, which may justify their lower flow values since a sealer with the same component also showed minimal flow ^18^.

Following ISO 6876^17^the endodontic sealer solubility must not exceed 3%. In this study, no tested sealers showed values close to recommended standards, although no significant differences were detected between them. These results agree with Estrela et al. ^19^, which demonstrated higher solubility values, above 10%, for ready-to-use sealers, and with Quaresma et al. ^16^. So, it is valid to deduce that higher solubility occurred due to the mass proportion of tricalcium silicate in the composition of the sealers, as well as the ultrafine size of their hydrophilic particles, which can increase the surface area and thereby allow greater contact between the liquid and the tested sealers ^16^ and also due elevated sealer’s setting time, as materials with long setting times are more susceptible to dissolution ^20^.

Calcium silicate-containing sealers can release calcium and hydroxyl ions after hydration because of the formation of portlandite which is calcium hydroxide ^21^. All sealers evaluated released both ions. By the way, BC showed higher pH values at the initial time, corroborating with a previous study ^3^, CEO 2 presented the same behavior, which can be explained by the presence of calcium chloride in its composition ^22^. AHPB presented higher values at 168h, unlike Souza et al. ^5^, who reported a decrease in pH sealer over time. AHPB demonstrated low alkalinization, which can be attributable to different percentages of calcium silicates and calcium aluminates in its composition ^23^. Besides, the difference between CEO 1 and CEO 2 pH during the experimental time, a decrease at 3h and 168h and a rise at 24h can be justified by Park et al. ^24^ findings that also reported a variation in pH values by time.

In general, no significant difference among all sealers at all times, except at 168h for CEO 1, which released more calcium ions than CEO. The presence of calcium chloride in CEO 2 composition can explain these results once this component promotes the reduction of setting time, not allowing a proper calcium ion release ^15^ ^,^ ^20^. The release of calcium ions depends on several factors, such as size, density, distribution of the mineral particles, and the network structure of the hydrated sealer matrix (the calcium silicate hydrate phase), which is responsible for water sorption, solubility, and water permeability ^25^. Therewith, since the ionic dissociation is influenced by sealer chemical composition, it is reasonable to consider the variation of calcium ions released by BC over time were higher at 3h, lower at 24h, and raiser at 72h, because of its composition, especially due presence of iron oxide in it dispersing agent, since the ions release is inversely proportional to the amount of iron oxide present in sealer’s composition ^25^.

The lowest push-out values were found in the presence of both experimental sealers concerning BC and AHPB. These results agree with Primus et al. ^2^, who also observed less than 10 MPa of bond strengths to dentin. In addition, cohesive failures were predominant for all sealers, these results corroborate with a previous study ^22^ once they documented the bioactive material's ability to promote biomineralization between dentin and sealer interface, suggesting a chemical bonding. 

The physicochemical properties of premixed endodontic sealers depended on their chemical composition and environmental conditions when confined within the root canal. In addition, these properties can influence/modify the host response and repair process directly related to the success of endodontic treatment. A limitation of this study lies in the absence of in vivo experiments that could verify the biological properties of the tested sealers. Further in vivo and clinical research are required for extensive clinical use.

## Conclusion

Based on the utilized methodology and the results obtained, CEO 1 and CEO 2 exhibited physicochemical properties similar to endodontics sealers already available. 
